# Arrhythmia risk stratification of patients after myocardial infarction using personalized heart models

**DOI:** 10.1038/ncomms11437

**Published:** 2016-05-10

**Authors:** Hermenegild J. Arevalo, Fijoy Vadakkumpadan, Eliseo Guallar, Alexander Jebb, Peter Malamas, Katherine C. Wu, Natalia A. Trayanova

**Affiliations:** 1 Institute for Computational Medicine and Department of Biomedical Engineering, Johns Hopkins University, Baltimore, Maryland 21218, USA; 2 Welch Center for Prevention, Epidemiology, and Clinical Research, Department of Epidemiology, Johns Hopkins Bloomberg School of Public Health, Baltimore, Maryland 21287, USA; 3 Division of Cardiology, Department of Medicine, Johns Hopkins Medical Institutions, Baltimore, Maryland 21287, USA

## Abstract

Sudden cardiac death (SCD) from arrhythmias is a leading cause of mortality. For patients at high SCD risk, prophylactic insertion of implantable cardioverter defibrillators (ICDs) reduces mortality. Current approaches to identify patients at risk for arrhythmia are, however, of low sensitivity and specificity, which results in a low rate of appropriate ICD therapy. Here, we develop a personalized approach to assess SCD risk in post-infarction patients based on cardiac imaging and computational modelling. We construct personalized three-dimensional computer models of post-infarction hearts from patients' clinical magnetic resonance imaging data and assess the propensity of each model to develop arrhythmia. In a proof-of-concept retrospective study, the virtual heart test significantly outperformed several existing clinical metrics in predicting future arrhythmic events. The robust and non-invasive personalized virtual heart risk assessment may have the potential to prevent SCD and avoid unnecessary ICD implantations.

## Abstract (web summary)

Sudden arrhythmic death is a leading cause of mortality, however approaches to identify at-risk patients are of low sensitivity and specificity. Here, the authors develop a personalized approach to assess arrhythmia risk in post-infarction patients based on cardiac imaging and computational modelling that significantly outperforms existing clinical metrics.

## Graphical abstract

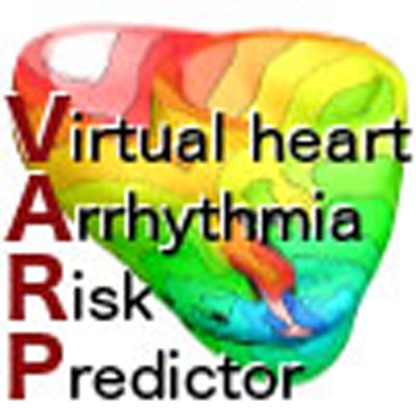

Sudden cardiac death (SCD) is a leading cause of death in the industrialized world^1^. A large proportion of SCDs result from ventricular arrhythmias—abnormal uncoordinated heart rhythms—particularly among patients with prior heart damage from myocardial infarction (MI). For patients at high risk for SCD, mortality is reduced by the prophylactic insertion of implantable cardioverter defibrillators (ICDs)^2^. To determine the level of SCD risk, clinical cardiology practice relies on a ‘one-size-fits-all' metric representing a global reduction in ventricular function: the left ventricular ejection fraction (LVEF)<35% metric^3^. Mechanistically, in MI, arrhythmia results from the heterogeneously distributed infarcted tissue, which can promote the initiation and maintenance of electrical instability^4^. Global LVEF poorly reflects these mechanistic factors^5^ and, hence, its use to determine the level of SCD risk and stratify patients for ICD implantation results in a low rate of appropriate ICD device therapy, only 5% per year^6^. Thus, many patients are exposed to ICD risks—infections, device malfunctions and inappropriate shocks—without deriving any health benefit^7^,^8^,^9^. Further, the LVEF metric only targets a relatively small subgroup of individuals at high risk for SCD, failing to identify the majority of SCD victims. Thus, inadequate SCD risk assessment poses a large public health and socioeconomic burden. Development of accurate non-invasive means of SCD risk stratification is a paramount unmet clinical need.

Here we present the proof of concept of a novel targeted approach to determine the risk of SCD in MI patients. The approach is based on cardiac imaging and computational modelling, and is personalized to each patient. To assess SCD risk, we construct a three-dimensional (3D) computer model of MI patient's individual heart from the clinical magnetic resonance imaging (MRI) data. The heart model incorporates the patient's ventricular geometry and MI structural remodelling as well as electrical functions from the sub-cellular to the organ. Thus, the interplay between abnormal myocardial structure and electrical instability in the heart that predisposes to SCD can be directly assessed. In each heart model, we conduct a virtual multi-site delivery of electrical stimuli from ventricular locations at different distances to remodelled tissue so that the patient's heart propensity to develop infarct-related ventricular arrhythmias can be comprehensively evaluated. We term this non-invasive SCD risk assessment approach VARP, a Virtual-heart Arrhythmia Risk Predictor. In a proof-of-concept study, we assess the predictive capability of the VARP approach as compared with that of other clinical metrics. Our results demonstrate that VARP significantly outperforms clinical metrics in predicting future arrhythmic events. The robust and non-invasive VARP approach may thus have the potential to prevent SCD and avoid unnecessary ICD implantations in post-infarction patients.

## Results

### The VARP approach

**Figure 1:**
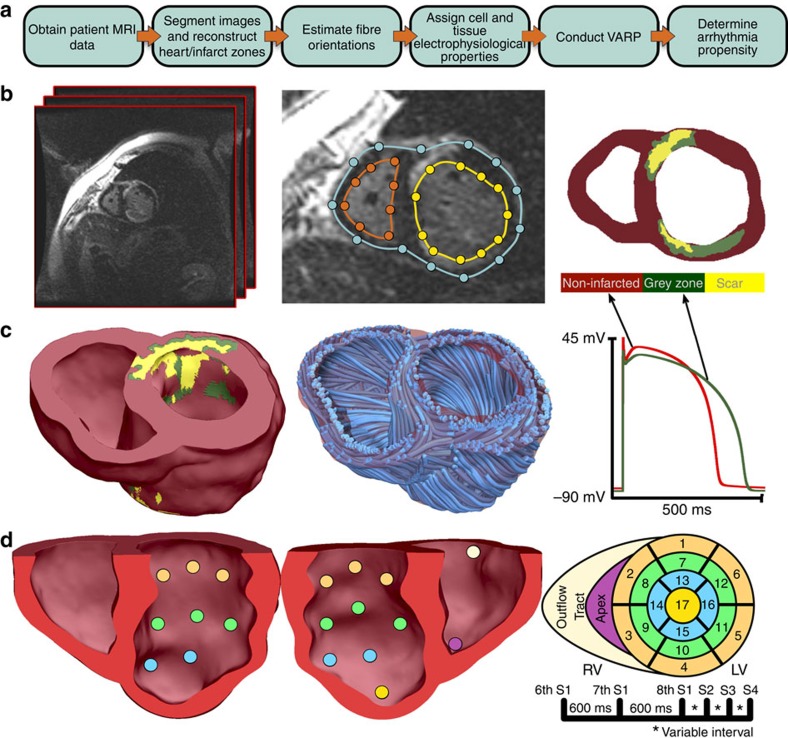
VARP methodology. (**a**) Flow chart summarizing the VARP protocol. (**b**) Contrast-enhanced cardiac MRI stack (left), with landmark points and splines delineating the endocardial and epicardial surfaces (middle), respectively, and the resulting ventricular segmentation (right) into non-infarcted myocardium, grey zone and scar. (**c**) High-resolution ventricular structure model (left) with estimated fibre orientations (middle). Although fibre orientation is assigned to each finite element in the computational mesh, a tractography approach is used here to visualize the general fibre orientation. Action potential traces from the non-infarcted myocardium (red) and grey zone (green) are in right panel. (**d**) VARP pacing sites on the endocardial surface of the ventricles (left panels) and a corresponding colour schematic (right) of the myocardial wall segments (numbered), as per the American Heart Association nomenclature, in which these sites are located. The train of pacing pulses is shown on the bottom right. Additional detail is provided in Methods.

A flow chart of the processes that comprise VARP is presented in Fig. 1a. First, 3D patient-specific ventricular wall geometry is reconstructed^10^ from the contrast-enhanced clinical MRI (Fig. 1b). Pixels in the ventricular myocardium are classified as non-infarcted or infarcted tissue based on signal intensity. Previous research^11^ has indicated that the presence of infarct border zone (termed also grey zone (GZ) because of its intermediate signal intensity in clinical MRI) contributes to arrhythmia propensity, thus pixels belonging to infarcted tissue are further sub-classified as scar or GZ. The reconstructed geometrical model of the infarcted ventricles is presented in Fig. 1c, left. Next, fibre orientations are assigned (Fig. 1c, middle); they are important determinants of electrical wave propagation in the heart^12^. Fibre orientations cannot be currently acquired from clinical MRI, therefore we estimate them using our novel geometry-driven rule-based approach^13^. Once the ventricular structure model is complete, region-specific cell and tissue electrophysiological (EP) properties are assigned to the finite elements in the model. Scar elements are considered electrically non-conductive. Myocytes in non-infarcted tissue and GZ are assigned human action potential dynamics, with GZ myocytes exhibiting remodelled ion channel kinetics resulting in action potentials of extended duration (Fig. 1c, right). GZ tissue conductivities are also remodelled, reflecting disease-induced changes in cell-to-cell connections.

The next step (Fig. 1d) is to electrically stimulate (pace) the virtual heart model from a number of bi-ventricular locations. Each stimulation is an attempt to elicit a re-entrant arrhythmia triggered from a site positioned differently with respect to remodelled tissue. In each patient-derived heart model, stimuli are delivered (Fig. 1d) at 17 locations in the left ventricle (LV), one in each AHA segment^14^, and at the apex and near the outflow tract of the right ventricle; the pulse train given at each site is shown in Fig. 1d. The response of the virtual heart to stimulation is calculated using a validated simulation approach^15^. A patient is classified as being at risk for SCD if arrhythmia is elicited from at least one of the 19 pacing locations (that is, a positive VARP test).

### Assessing the predictive capabilities of VARP

The predictive capability of the VARP approach was evaluated retrospectively in a proof-of-concept study using the data from 41 patients with prior MI and LVEF<35%. The patients were chosen randomly from the 136 patients enrolled before April 2009 in the CMR-Prospective Observational Study of Implantable Cardioverter Defibrillators (CMR-PROSE-ICD)^11^,^16^,^17^. We chose a cohort that was balanced between patients with (*n*=21) and without (*n*=20) arrhythmic events; the rationale for this choice is provided in Methods. All patients underwent implantation of clinically indicated ICDs; contrast-enhanced MRIs were obtained pre-ICD implantation. Patients were followed for the primary end point of appropriate ICD firing due to ventricular arrhythmia or cardiac death. Follow-up time averaged 4.8±2.9 years. Supplementary Tables 1 and 2 summarize the baseline patient characteristics and Supplementary Table 3 presents the VARP outcomes; further details are provided in Methods below. For this patient cohort, VARP predictive capabilities were compared to the current clinical metric, LVEF, as well as to other existing clinical metrics that have been used to predict arrhythmic risk, such as GZ volume^18^, scar volume^19^ and LV mass^20^. Furthermore, 32 of the 41 patients in the cohort underwent, at the time of ICD implantation, an invasive procedure, termed clinical EP testing^21^, a non-routine clinical means of assessing arrhythmia propensity (see Methods for detail). For these 32 patients, VARP assessment was also compared with the outcome of clinical EP testing. All VARP tests were conducted by investigators who were blinded to clinical outcomes.

**Figure 2:**
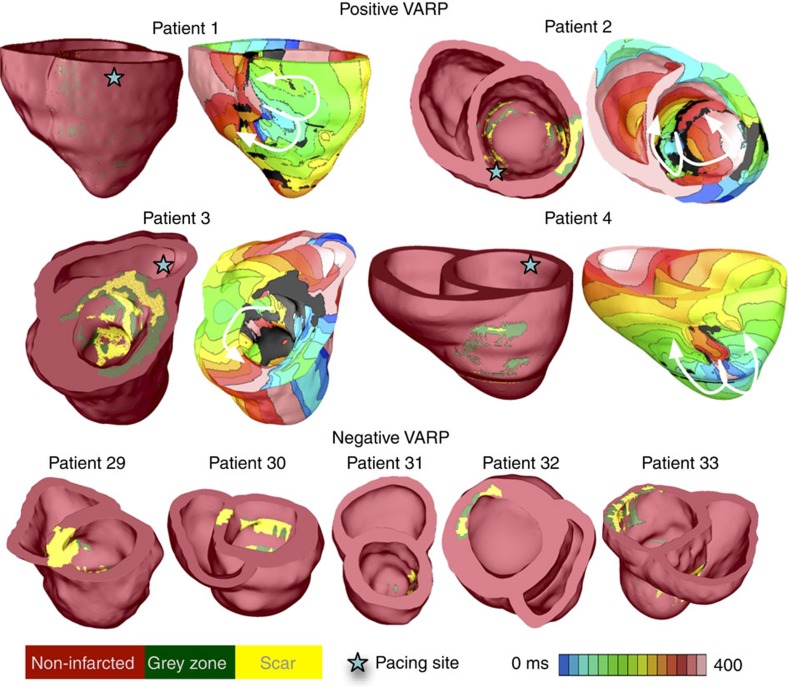
Illustrative examples of VARP results for 9 of the 41 personalized heart models. Shown is the induced arrhythmia in four hearts (top), for which geometrical models are presented together with electrical activation isochronal maps, obtained following pacing from the site indicated by the star. White arrows represent the direction of propagation of the re-entrant arrhythmias. All induced arrhythmias were monomorphic ventricular tachycardias. The geometrical models of the five hearts, in which no arrhythmia was induced from any pacing site, are shown at the bottom.

**Figure 3:**
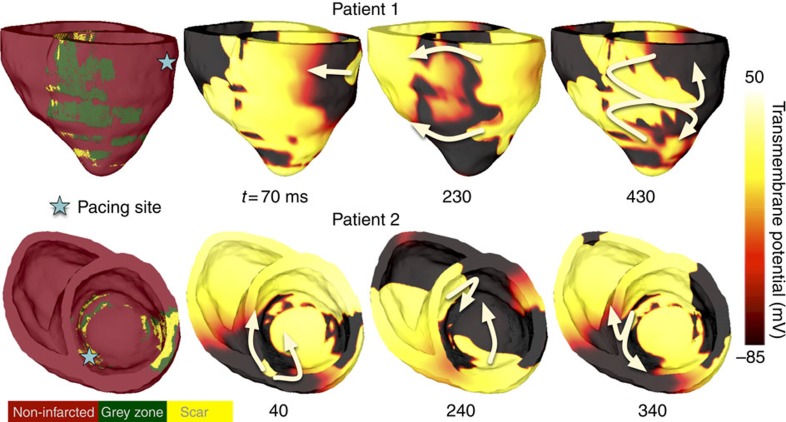
Initiation of ventricular tachycardia in patients 1 and 2. Shown are patient heart geometries and transmembrane potential maps at three time points. White arrows show direction of propagation. The time instant below each map is counted from the delivery of the last pacing stimulus. In patient 1, conduction block occurs in a GZ region located in the anterior portion of the ventricles. The wavefront propagates around that GZ region and forms a figure-of-8 re-entrant circuit. In patient 2, unidirectional block occurs in GZ region located in the septum. The wavefront re-enters via an isthmus of excitable myocardium and forms a re-entrant circuit that eventually anchors to intramural scar. See Supplementary Movies 1 and 2 for corresponding movies of the VT initiation and resulting VTs.

Figure 2 presents nine reconstructed patient heart models; arrhythmia was induced in four (see Supplementary Figs 1 and 2 for all models). Shown are electrical activation maps obtained in each model following VARP, with pacing from the location indicated. The activation maps exhibit the wave rotational sequence characteristic of re-entrant arrhythmias. Re-entrant wave fronts often propagate through isthmuses in the scar, also evident from the transmembrane potential maps (Fig. 3; Supplementary Movies 1 and 2). In the five remaining models in Fig. 2, no arrhythmia was induced from any pacing site, despite the presence of infarcted tissue (the remaining non-inducible models are shown in Supplementary Fig. 3).

Statistical analysis, the detail of which is provided in Methods, demonstrated that a positive VARP test was significantly associated with the primary end point, with a fourfold higher arrhythmia risk than patients with negative VARP test (*P*=0.03, conditional logistic regression; Table 1). The comparison of VARP with the routine clinical risk metric, LVEF, as well as with the other clinical risk predictors (GZ volume, scar volume and LV mass), revealed that only VARP outcome was significantly associated with arrhythmic risk in this cohort (Table 1). When only appropriate ICD shock was used as a secondary end point, the hazard ratio for VARP increased from 4.05 to 5.0 (95% confidence interval 1.15–21.9, *P*=0.032, conditional logistic regression).

Among patients who had both VARP and invasive clinical EP testing (*n*=32), the hazard ratio for VARP was 10.4 (95% CI 1.4–79, *P*=0.02, conditional logistic regression) versus 1.7 (95% CI 0.6–4.8, *P*=0.35, conditional logistic regression) for clinical EP testing. For the appropriate shock end point, the hazard ratio for VARP remained significant at 8.60 (95% confidence interval 1.12–66.09, *P*=0.04, conditional logistic regression) versus 2.60 for clinical EP testing (95% confidence interval 0.72–9.32, *P*=0.14, conditional logistic regression). Clearly, the non-invasive nature of VARP offers an additional advantage over clinical EP testing, which entails risks of vascular access, sedation and induction of ventricular arrhythmias requiring defibrillation in already tenuous cardiomyopathy patients.

## Discussion

This study demonstrated, in a 41-patient cohort, that the VARP non-invasive personalized virtual heart approach is appreciably superior in predicting arrhythmia risk associated with MI, as compared with the current clinical metric, LVEF, as well as to other existing non-invasive clinical metrics and the invasive clinical EP testing, a non-routine clinical means of assessing arrhythmia propensity. The superiority of the approach is rooted in its ability to comprehensively evaluate the arrhythmogenic propensity of the MI substrate as probed by triggers acting at ventricular locations of different geometrical position with respect to remodelled tissue.

As a proof-of-concept study, the evaluation of the novel targeted approach for determining arrhythmia risk was done in a small number of patients. Should the predictive capability of the approach be demonstrated in larger studies, VARP has the potential to radically change the process of SCD risk assessment and patient selection for prophylactic ICD implantation. The approach could eliminate many unnecessary ICD implantations and their associated complications (infections, device malfunctions and inappropriate shocks), benefiting innumerable patients. Importantly, the methodology could be applied to patients with prior MI but preserved LVEF >30–35%, who could also be at significant risk for arrhythmia because of their remodelled myocardium, but are generally not targeted for therapy under current clinical recommendations^22^. Indeed, because current guidelines for ICD placement target low LVEF patients who constitute only one-third of SCD victims^23^, VARP has the potential to identify increased SCD risk in a much larger number of at-risk patients. We also envision that patients could be re-imaged and VARP repeated to account for changes in arrhythmia susceptibility over time as the diseased heart remodels.

The VARP approach is easily extendable to patients with non-ischaemic cardiomyopathy, where myocardial structure incorporates a distributed scar. Finally, because our simulation platform represents processes from the molecular to the whole organ, it can be potentially modified to input patient-specific genetic and pathophysiological data. Thus, we envision that the approach could be significantly broadened to stratify SCD risk for cardiac diseases of various etiologies, and also combined into multi-factor risk models on a patient-specific basis.

This is the first study to develop a significant number of patient-derived computational models of the heart and apply them to address a clinical need. Previous cardiac EP models have been limited to very small sample sizes or simplified geometries, and had been used to examine arrhythmia mechanisms. This study makes a leap forward in integrating image-based computational modelling of the heart into heart disease diagnosis and treatment. We believe that computer modelling is poised to transform areas of medicine and serve as a vehicle to advance personalized approaches to human health.

## Methods

### Patient-specific geometrical model construction

Patient-specific geometrical models of 3D ventricular structure were reconstructed from the contrast-enhanced MR images. For each patient, the myocardial boundaries in the two-dimensional slices in the MRI stack were contoured with cubic splines fit through landmark points manually identified using the software ImageJ^24^ (Fig. 1b, middle). From the landmark points, the patient-specific 3D ventricular wall geometry was reconstructed using a methodology based on variational implicit functions interpolation developed and validated previously by our team^10^ (Fig. 1c, left). To represent the personalized geometry of the infarct in each ventricular geometry reconstruction, the myocardial regions in the two-dimensional slices of the MR image were classified as infarcted and non-infarcted areas by means of signal thresholding performed in CineTool (General Electric Healthcare). Each infarct region was further classified into scar and GZ using a full width half maximum approach validated previously by our team^11^ (Fig. 1b, right). The 3D geometries of the infarct zones were reconstructed using a shape-based interpolation method^25^, and merged with the corresponding ventricular geometry reconstruction (Fig. 1c, left).

Next, the 3D volumetric finite element computational mesh of each infarcted heart was generated using an octree-based approach for image-based mesh generation developed by our team^26^. The meshing technique is automatic, and produces boundary-fitted, locally refined and smooth conformal meshes. Each finite element ventricular mesh had an average resolution of 350 μm; ventricular models thus comprised of ∼4 million nodes. The choice of finite element size was dictated by the need to resolve wavefront propagation in the simulations while simultaneously minimizing computational expense, and validated by our team^27^,^28^.

Finally, fibre orientations were assigned to each ventricular computational mesh using an efficient rule-based approach developed and validated by our group^13^. Fibre orientations were assigned in each model on the basis of the individual geometry of the ventricles. The fibre orientation methodology used the Laplace–Dirichlet method^29^,^30^ to define transmural and apicobasal directions at every point in the ventricles. It then employed bi-directional spherical linear interpolation to assign fibre orientations based on a set of fibre orientation properties (rules) derived from a large amount of histological and diffusion tensor MRI data. After fibre orientations were assigned to all elements in the ventricular mesh, the corresponding ‘masks' of infarct scar and GZ were superimposed.

Altogether, reconstruction of each patient heart took ∼8 h.

### Electrophysiological modelling

Once the 3D finite element ventricular mesh was generated, cell and tissue EP properties were assigned to the three regions: scar, GZ and non-infarcted tissue. All finite elements that belonged to the scar region were considered electrically non-conductive. Finite elements that belonged to non-infarcted tissue and GZ were assigned human ventricular cell action potential dynamics^31^. Modifications to the ionic model based on experimental recordings were implemented to represent EP remodelling in the GZ. Specifically, patch-clamp studies of cells harvested from the infarct border zone have reported a 62% reduction in peak sodium current^32^, 69% reduction in L-type calcium current^33^ and a reduction of 70 and 80% in potassium currents *I*_Kr_ and *I*_K_, respectively^34^. As a result, the GZ action potential was characterized by a longer duration, decreased upstroke velocity and decreased peak amplitude compared with those in the non-infarcted myocardium (360 versus 310 ms, 6.7 versus 11.6 V s^−1^ and 20 versus 35 mV, respectively), similar to what has been previously reported^35^,^36^.

Since no significant change was found in the density of connexin-43, the gap junction protein responsible for cell-to-cell electrical communication, in non-infarcted myocardium of infarcted hearts^37^, we used the conductivities of normal tissue^38^,^39^ in these regions. These conductivities were further adjusted using a systematic approach^40^ to match human myocardium conduction velocity measured in experimental studies^41^,^42^,^43^,^44^. The values of the non-infarcted tissue conductivities used in this study were 0.255 and 0.0775 S m^−1^ in the longitudinal and transverse directions, respectively.

Tissue in the GZ region was characterized with a 90% decrease in transverse conductivity to reflect connexin-43 remodelling in the infarct border zone^45^. No additional change in fibre orientation was implemented in the GZ since evidence of the degree of the potential change in fibre orientation in the GZ is lacking. We assessed the impact of this potential uncertainty in fibre orientation in the GZ. On the basis of the analysis in the study by Bayer *et al*^13^, we found that, given that GZ volume is on average 11.6% of total ventricular volume (Supplementary Table 1), up to 25° change in fibre orientation in GZ would result in up to 2 ms change in activation time in the GZ. Thus, we concluded that the consequences of the uncertainty in GZ fibre orientation would be minimal.

### Simulation of electrical activity and numerical aspects

The propagation of electrical activity in a virtual heart was simulated by solving, using the finite element method, a reaction-diffusion partial differential equation representing the spread of current in the ventricular myocardium, together with the ordinary differential and algebraic equations representing myocyte membrane dynamics at each node in the mesh^27^. The system of equations was solved with a time step of 25 μs. Simulations of electrical activity in the patient-specific heart models were executed in a monodomain representation of the myocardium using the software package CARP (CardioSolv LLC) on a parallel computing system. The software utilizes sophisticated solver techniques that have been optimized to ensure the high levels of accuracy, stability and efficiency in obtaining solutions on the large computational meshes necessary to model electrical behaviour in human hearts^46^,^47^. Using 40 processors, simulation run time was ∼1 h for each second of simulated activity. Solutions to EP problems (for example, arrhythmogenesis and defibrillation in the rabbit heart) using this software have been experimentally validated in a number of publications from our team^48^,^49^,^50^ and used in mechanistic human arrhythmia studies^51^.

### Validation of modelling of post-infarction arrhythmias

The approach to construct a model of the infarcted ventricles by thresholding the infarct into scar and (homogeneous) GZ, as done in the present study, has been recently validated with experimental data. Deng *et al*.^15^ used sock epicardial data for infarct-related ventricular tachycardia (VT), obtained from *in vivo* swine hearts, and demonstrated that ventricular models reconstructed from MRI data of the corresponding hearts were able to predict fairly accurately the morphology of each VT re-entrant circuit and its organizing centre (for example, isthmus). This indicates that small heterogeneities in GZ, the Purkinje system and additional regional EP heterogeneities play a secondary role, with the geometrical morphologies of scar and GZ, as well as the representation of different EP properties in non-infarcted tissue and GZ being primary, in determining the inducibility of a given VT and the location of its organizing centre. This is consistent with the findings by Arevalo *et al*.^52^, where a parameter sensitivity analysis of the GZ model representation was conducted. The study found that the inclusion of small scar heterogeneities in a physiological density did not alter inducibility of infarct-related VT. These studies provide a justification for the EP modelling approach undertaken in this study.

### VARP protocol

Each patient-derived ventricular model was subjected to pacing from multiple locations in an attempt to elicit re-entrant arrhythmias, thus assessing the potential of the disease-remodelled ventricles to cause degeneration of electrical signal propagation into arrhythmic activity following premature beats that originate at different locations in the heart. For SCD risk stratification, as discussed in a recent editorial^53^, the site or mode of stimulation of the heart and the resultant different VT morphologies induced are of little importance—what matters is whether or not arrhythmia is induced. Thus, the VARP protocol was designed to take maximum advantage of the capabilities of a validated simulation platform. Each virtual heart was paced from 19 different locations on the ventricular endocardium (Fig. 1d), two right ventricular endocardial sites (one at apex and another from a central location near the outflow tract) plus 17 pacing sites on the LV endocardium, one in each AHA segment^14^. The rationale for choosing a large number of pacing sites was based on clinical studies, which have shown a positive correlation between the number of pacing sites and inducibility of ventricular arrhythmia^54^,^55^. The distribution of pacing sites throughout the LV ensured that the protocol covered a large range of possibilities for potential sites at which ectopic foci could emerge and captured all the possible arrhythmias that could arise from the given infarct morphology, thus fully assessing the arrhythmogenic propensity of the substrate. In a previous computational study^52^, we showed that further increasing the number of pacing sites does not uncover more unique VTs. All pacing sites were assigned in the model automatically using an approach described previously^56^.

The pacing pulse train was generally similar to pacing trains delivered in standard clinical protocols^16^. It consisted of eight pacing stimuli (S1) at a cycle length of 600 ms. A premature stimulus (S2) was delivered 300 ms after S1. If S2 did not result in the generation of re-entrant arrhythmia, the S1–S2 interval was shortened, in 10-ms steps, until arrhythmia was induced or the S2 failed to capture the tissue. If arrhythmia was not induced, an additional S3, and if necessary S4, was delivered in the same manner as S2 (initially delivered 300 ms after previous stimulus, and then shortened until arrhythmia was induced or the stimulus failed to capture). In all simulations, the size of the pacing electrode was 1 × 1 × 1 mm, injecting transmembrane current. Simulations were monitored to ensure that in each case an excitation wave was initiated and propagated away from the pacing location. The re-entrant arrhythmia periods among all patients and all pacing sites in the 41 patients were in the range 276–445 ms, which is consistent with reported human VT cycle lengths^57^.

Overall, 779 whole-heart simulations were performed (arrhythmia induction tested in 41 hearts from 19 pacing sites), rendering the current simulation study the largest cardiac simulation study performed thus far. To ensure computational tractability of the study, each simulation run calculated ∼7 s of electrical activity in the ventricles (corresponding to 7 h of execution time), the first 5 s of which was the pacing protocol, and the remaining 2 s represented the post-pacing period used to detect the presence of arrhythmia. In this 2-s time interval, when activity was present after the cessation of pacing, three scenarios were observed: sustained arrhythmia, unsustained arrhythmia and an incomplete re-entry (single beat). We made a choice to count all episodes of unsustained and sustained arrhythmia as positive outcome in regards to inducibility (the 1 s criterion separated the unsustained/sustained arrhythmias from the single beat). Thus, VARP outcome was classified as positive if re-entrant arrhythmia that persisted for >1 s was elicited. The rationale for the choice to count both unsustained and sustained arrhythmias was the following: we observed that in the cases of unsustained arrhythmia, often a small change in the size of an isthmus or other feature of the infarct zone converted an unsustained arrhythmia into sustained. Given that there are uncertainties in the image processing involved in model construction, we made a choice to err on the side of increased sensitivity of the VARP approach.

### Image processing and simulation tools

The image processing software ImageJ is available from http://imagej.nih.gov/ij/. For grey-level thresholding, the software CineTool was used here (GE Medical Systems). Computational meshes are generated using the software Tarantula (CAE Software Solutions). The human ventricular ionic model by ten Tusscher *et al*. is freely available from the repository CellML (https://www.cellml.org/). The rule-based approach to assign fibre orientations in the computational mesh can be reproduced from the original publication^13^, which presents a set of algorithms and subroutines that can be easily implemented. The electrophysiology simulations were executed using the software package CARP (CardioSolv, LLC). The simulations can also be executed using the open-source software CHASTE (http://www.cs.ox.ac.uk/chaste/). The patient MRI images used to construct the personalized heart models are available on request and on approval of Johns Hopkins Institutional Review Board.

### Evaluating VARP predictive capabilities with patient data

The predictive capability of VARP in stratifying SCD risk was evaluated retrospectively using data from 41 patients with prior MI and LVEF<35% chosen randomly from the 136 patients enrolled in PROSE-ICD clinical trial^16^,^17^ with MRI scans acquired before April 2009. All patients underwent implantation of clinically indicated ICDs. We chose a cohort that was balanced between patients with (*n*=21) and without (*n*=20) arrhythmic events. Of the 21 patients who reached the primary end point, 18 had appropriate shocks and 3 had cardiac death. Cardiac death was classified as a primary end point to minimize under detection of potentially arrhythmic events in the low risk group. Appropriate shock only was assessed as a secondary end point.

The sample size was chosen following the rule of thumb that survival data need to include at least 10 outcomes of each kind per independent variable, for sufficient confidence in the results^58^. The sample size also ensured computational tractability of the simulations, given the run times indicated above. Specifically, since only small numbers of patients with ICDs have arrhythmia events (∼5% annually)^6^, if the patient cohort was not balanced between patients with and without arrhythmia, the majority of the whole-heart models would have been not inducible for arrhythmia. The small number of non-inducible cases in the cohort would have then prevented us from assessing statistically the arrhythmia risk predictive capability of VARP. We also could not increase the number of patients in the cohort above 41 because of issues of computational expense, as outlined in the section above.

Patient characteristics for the nested case–control group and the entire cohort are presented in Supplementary Tables 1 and 2 in Supplementary Information, respectively. For the patients who underwent VARP, mean±s.d. age was 61.8±11.1 years. Participants were 78% men and 82% Caucasian. Fifty-one percent of the patients reached primary end point at a mean of 2.8±1.8 years after enrolment. The mean follow-up time in patients without events was 6.8±2.2 years. There was no significant difference in risk factors or medications between patients who did and did not have events.

All patient images used here are from the CMR-PROSE-ICD study^11^,^59^. Patients underwent contrast-enhanced short-axis MRI with a 1.5-T scanner (Signa CV/i, GE Healthcare Technologies, Waukesha, Wis, or Avanto, Siemens, Erlangen, Germany) pre-ICD implantation. Each patient's MRI stack consisted of 10–14 contiguous short-axis slices. The MRI was gated and the reconstructed geometry was diastolic. Late gadolinium-enhanced images were acquired 15–30 min after a total injection of 0.2 mmol kg^−1^ gadodiamide (Omniscan, GE Healthcare Technologies) with an inversion recovery fast gradient-echo pulse sequence. Imaging parameters were as follows: TR 5.4-8.3 ms, echo time 1.3–3.9 ms, average in-plane spatial resolution 1.5 × 2.4 mm, 8-mm slice thickness, 2-mm gap and inversion time (TI) adjusted to null the signal of normal myocardium. All imaging acquisition parameters were standardized and pre-specified, and have been published previously^11^,^17^. The segmentation signal intensity threshold values used for this study were determined using a semi-automatic full width half maximum approach developed previously by our team^11^; reproducibility of segmentation was also assessed in that publication. The protocol was approved by the Johns Hopkins Hospital Institutional Review Board, and all patients provided written informed consent.

The clinical EP testing data are also from the CMR-PROSE-ICD study^11^,^59^. The EP testing protocol consisted of three extrastimuli at two different drive cycle lengths delivered from the right ventricular apex alone (ICD, *n*=30) or the right ventricular apex and outflow tract (EP study, *n*=2).

### Statistical analysis

As specified above, the 21 cases and 20 controls were selected at random among 41 participants with events and 95 participants without events in the CMR-PROSE-ICD cohort. Baseline characteristics for the entire cohort (Supplementary Table 2) and the case–control group (Supplementary Table 1) were summarized as median together with interquartile range for continuous variables or proportions for categorical variables. The characteristics were compared between the two groups using two-tailed rank-sum test^60^ or Fisher's exact test^61^, as appropriate. Hazard ratios and corresponding 95% confidence intervals were calculated using conditional logistic regression models^62^. All statistical tests were performed using a significance level of *P*=0.05. Stata (StataCorp LP) was used to perform all analyses. All tests we utilized here were appropriate for the data.

## Additional information

**How to cite this article**: Arevelo, H. J. *et al*. Arrhythmia risk stratification of patients after myocardial infarction using personalized heart models. *Nat. Commun.* 7:11437 doi: 10.1038/ncomms11437 (2016).

## Supplementary Material

Supplementary Information Supplementary Figures 1-3 and Supplementary Tables 1-3

Supplementary Movie 1 VARP: Initiation of VT for Patient 1. The pacing site is shown in the corresponding image of the heart model in Figure 3. The movie starts at the delivery of the 7th S1 (t=3600ms) and shows the subsequent delivery of the 8th S1 (t=4200ms), S2 (t=4550ms), S3 (t=4820ms), and 1500ms of induced VT. The movie shows propagation through patchy infarct areas.

Supplementary Movie 2 VARP: Initiation of VT for Patient 2. The pacing site is shown in the corresponding image of the heart model in Figure 3. The movie starts at the delivery of the 7th S1 (t=3600ms) and shows the subsequent delivery of the 8th S1 (t=4200ms), S2 (t=4680ms), S3 (t=5020ms), and 1500ms of induced VT. The movie shows propagation through patchy infarct areas and reentry via an isthmus in the scar.

## Figures and Tables

**Table 1:** Hazard ratios for the primary end point.

Predictor	Hazard ratio (95% CI)	*P* value
VARP	4.05 (1.20–13.8)	0.03
LVEF	0.95 (0.90–1.01)	0.12
GZ volume	1.02 (0.98–1.06)	0.26
Scar volume	1.02 (0.99–1.04)	0.16
LV mass	1.00 (0.99–1.01)	0.98

CI, confidence interval; GZ, grey zone; LVEF, left ventricular ejection fraction.
