# Supplementary material for: Arrhythmia risk stratification of patients after myocardial infarction using personalized heart models
Source: Nat Commun. 2016 May 10;7:11437. doi: 10.1038/ncomms11437 (PMC4866040; doi:10.1038/ncomms11437)
Supplement: Supplementary Information — Supplementary Figures 1-3 and Supplementary Tables 1-3 [file ncomms11437-s1.pdf]

Supplementary Information

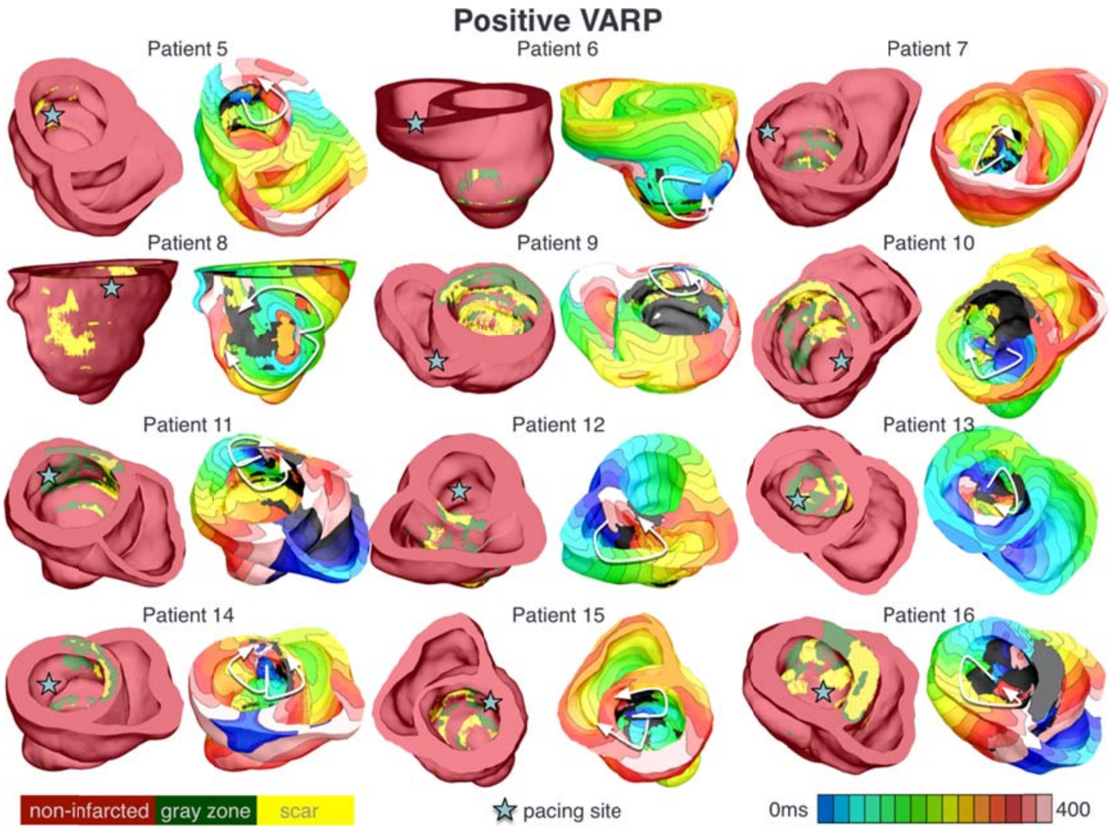

Supplementary Figure 1: VARP results for 12 of the 41 personalized heart models in which the test was positive. Geometrical models are presented together with electrical activation isochronal maps obtained following pacing from the site indicated by the star. White arrows represent the direction of propagation of the reentrant arrhythmias. All induced arrhythmias were monomorphic ventricular tachycardias (VTs).

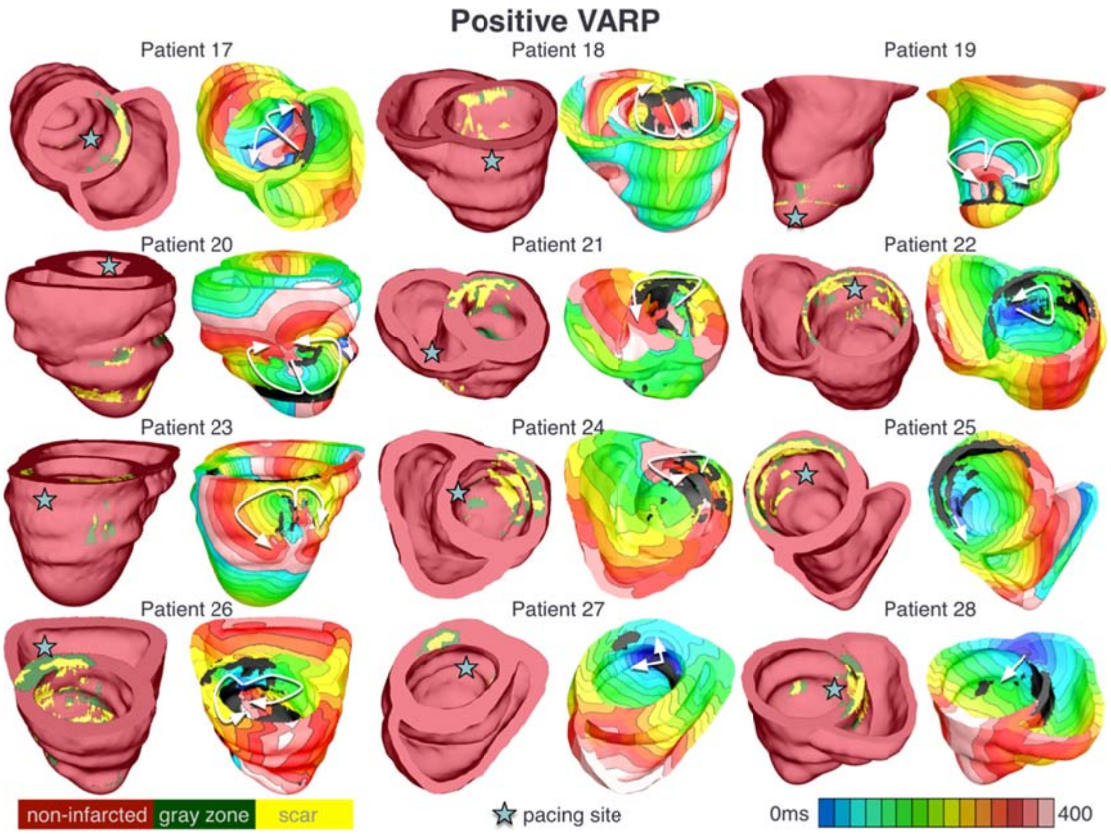

Supplementary Figure 2: VARP results for another 12 of the 41 personalized heart models in which the test was positive. Geometrical models are presented together with electrical activation isochronal maps obtained following pacing from the site indicated by the star. White arrows represent the direction of propagation of the reentrant arrhythmias.

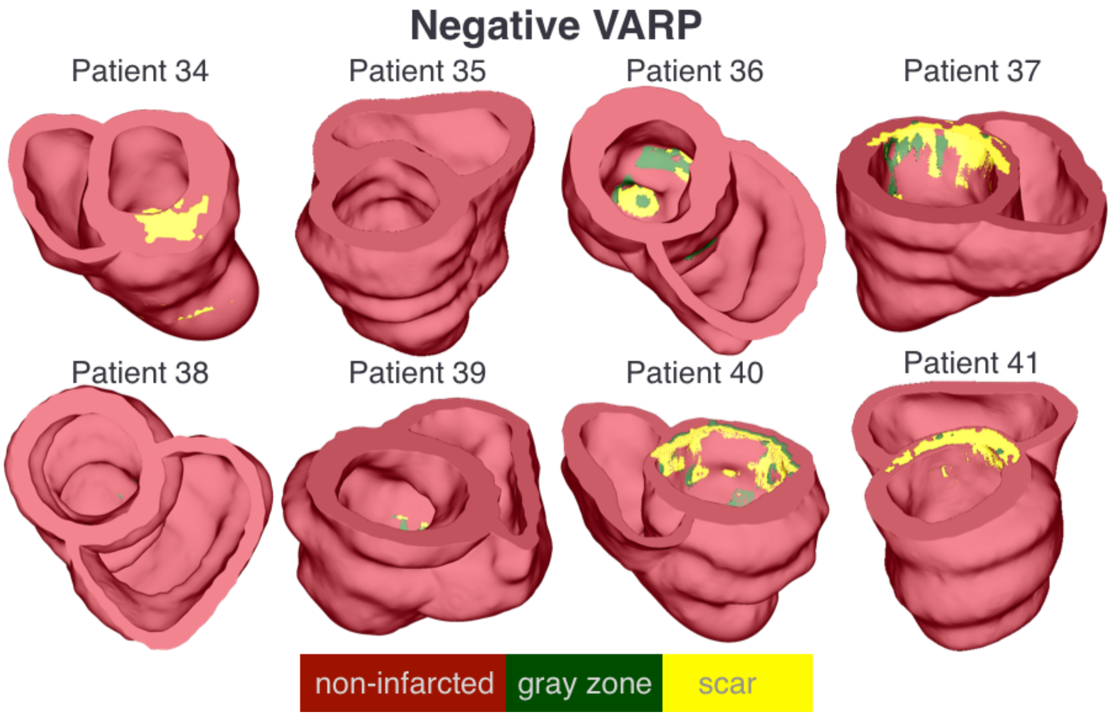

Supplementary Figure 3: Geometric models for 8 personalized heart models in which the VARP test was negative.

Supplementary **Table 1:** Baseline Characteristics of the VARP Cohort (Case- Control Group)

	**No endpoint (n=20)**	**Primary Endpoint (n=21)**	***p***
Male	16 (80%)	16 (76%)	1
Age	61 (IQR = 55 – 74)	62 (53 – 71)	0.71
Non-Caucasian	4 (20)	3 (14)	0.70
Ejection Fraction	26 (24 – 30)	20 (15 – 25)	0.09
**New York Heart Association functional class**			1
I	6 (30)	6 (28)	
II	8 (40)	9 (43)	
III	6 (30)	6 (29)	
Hypertension	16 (80)	13 (62)	0.31
Hypercholesterolemia	16 (80)	17 (81)	1
Diabetes	5 (25)	8 (38)	0.51
Nicotine Use	15 (75)	18 (86)	0.45
Beta Blocker	19 (95)	19 (90)	1
Ace Inhibitor or Angiotensin Receptor Blocker	17 (85)	20 (95)	0.34
Antiarrhythmics	2 (10)	4 (20)	0.66
Lipid Lowering	20 (100)	20 (95)	1
Spironolactone	2 (10)	4 (20)	0.66
Diuretic	10 (50)	14 (67)	0.35
Digoxin	5 (25)	3 (14)	0.45
Atrial Fibrillation	5 (25)	5 (24)	1
**CMR Characteristics**			
LV end-diastolic volume, mL	214 (177 – 252)	228 (196 – 286)	0.39
LV end-systolic volume, mL	155 (122 – 180)	169 (137 – 205)	0.14
LV mass, g	138 (113 – 191)	138 (115 – 155)	0.98
Total infarct mass, g	33 (19 – 43)	43 (30 – 53)	0.33
Gray Zone mass, g	13 (7 – 22)	16 (12 – 28)	0.30
Scar mass, g	18 (15 – 21)	21 (15 – 28)	0.30
Gray Zone/Total infarct mass, (%)	43 (35 – 50)	48 (39 – 50)	0.55
Scar/LV mass, (%)	15 (8 – 16)	15 (12 – 19)	0.53
Gray Zone/LV mass, (%)	10 (4 – 18)	12 (7 – 19)	0.51

Supplementary Table 2: Baseline Characteristics of Entire CMR-PROSE-ICD Cohort

	**No endpoint (n=95)**	**Primary Endpoint (n=41)**	***p***
Male	81 (85%)	35 (85%)	1
Age	59 (IQR = 54 – 69)	60 (53 – 71)	0.88
Caucasian	16 (17)	8 (20)	0.91
Ejection Fraction	25 (20 – 30)	25 (20 – 30)	0.43
**New York Heart Association functional class**			0.78
I	35 (37)	13 (32)	
II	33 (35)	14 (34)	
III	27 (28)	14 (34)	
Hypertension	62 (65)	31 (76)	0.32
Hypercholesterolemia	71 (75)	32 (78)	0.83
Diabetes	29 (31)	17 (41)	0.24
Nicotine Use	61 (64)	35 (85)	0.01
Beta Blocker	89 (94)	38 (93)	1
Ace Inhibitor or Angiotensin Receptor Blocker	82 (86)	38 (93)	0.39
Antiarrhythmics	7 (7)	5 (12)	0.35
Lipid Lowering	85 (89)	37 (90)	1
Spironolactone	20 (21)	8 (20)	1
Diuretic	48 (51)	28 (68)	0.06
Digoxin	14 (15)	6 (15)	1
Atrial Fibrillation	17 (18)	9 (22)	0.64
**CMR Characteristics**			
LV end-diastolic volume, mL	222 (183 – 266)	233 (192 – 294)	0.24
LV end-systolic volume, mL	159 (126 – 192)	175 (135 – 216)	0.14
LV mass, g	135 (114 – 180)	147 (118 – 179)	0.34
Total infarct mass, g	35 (22 – 46)	42 (28 – 52)	0.09
Gray Zone mass, g	14 (7 – 21)	17 (12 – 27)	0.03
Scar mass, g	19 (14 – 28)	22 (15 – 28)	0.31
Gray Zone/Total infarct mass, (%)	41 (32 – 48)	45 (37 – 51)	0.08
Scar/LV mass, (%)	15 (9 – 20)	16 (12 – 19)	0.47
Gray Zone/LV mass, (%)	10 (4 – 16)	12 (8 – 19)	0.04

Supplementary **Table 3:** VARP Pacing Sites, Numbered as Shown in Figure 1D, for which Arrhythmia was Induced (Patients 1-28).

Patient	Unsustained	Sustained
**1**	6,7,12,OT	4,9,15,16,17,apex
**2**	10	3,OT
**3**	1,apex	
**4**	1,9,14,15	apex
**5**	12	
**6**	3,4,5,7,12,17,apex,OT	6,15
**7**	1,3,14,15,17	
**8**		6,12,16
**9**	4,10,11,OT	2,7,8,9,14,15,17,apex
**10**	1,4,5,6,7,9,11,14,17	
**11**	1,5	4
**12**	17	5,7,10,11
**13**		2,7,13,OT
**14**	7	5,9,10,12,14,15,17,apex
**15**		1,3,6,12,16,apex
**16**		9
**17**		2,9
**18**		4,10
**19**		17
**20**	3	8,9,14,15
**21**		1,2,6,7,OT
**22**	1,2,7	
**23**	7	4,5,6,13,16,17
**24**	2	
**25**	11	
**26**	5,6,11,12,17,apex,OT	3,7,8,13
**27**	10	
**28**	3,9,OT	

Legend: OT – outflow tract
